# Variable hydrology and salinity of salt ponds in the British Virgin Islands

**DOI:** 10.1186/1746-1448-2-2

**Published:** 2006-02-27

**Authors:** Lianna Jarecki, Mike Walkey

**Affiliations:** 1 H. Lavity Stoutt Community College, Box 3097, Road Town, Tortola, British Virgin Islands; 2 Durrell Institute of Conservation and Ecology, University of Kent at Canterbury, Canterbury, Kent CT2 7NZ, UK

## Abstract

Caribbean salt ponds are unique wetlands that have received little scientific attention. They are common features of dry Caribbean coastlines, but they are threatened by rapid coastal development. We compared hydrology and salinity of 17 salt ponds in the British Virgin Islands. Ponds were mostly hypersaline (>50 ppt), and they exhibited dramatic salinity fluctuations in response to rainfall and evaporation. Individual ponds varied in their mean salinities and thus experienced different ranges of salinity. Differences in mean salinity appeared to be linked with hydrological characteristics. Hydrological variation ranged from permanently inundated ponds with direct sea connection to those fully isolated from the sea and retaining water only after rainfall. We characterized groups of ponds by their major hydrological characteristics, particularly their period of inundation and their degree of connection with the sea. The resulting classification appeared to reflect a continuum of increasing isolation from the sea, concurring with published geological records from salt pond sediments elsewhere. The patterns of variability and succession described here are applicable to salt pond management interests throughout the Caribbean.

## Background

Salt ponds are enclosed or mostly enclosed water bodies that occur within coastal mangrove wetlands. They are typically hypersaline, as defined by Hammer, 1986 [1], with water salinities typically in excess of 50 parts per thousand (ppt) [2]. Salt ponds and their surrounding mangrove forests, together known as "basin mangrove forests" [3], are the predominant type of coastal wetland in the Caribbean [4]. These wetlands provide important ecological services, including storm protection and flood mitigation, shoreline stabilization, erosion control, and retention of nutrients and sediments [5-7]. They also provide critical habitat and food resources for resident and migratory birds in the Caribbean [8].

Mangrove wetlands throughout the Caribbean are being replaced by coastal developments, and they are now considered to be one of the most threatened habitats on Earth [9]. Despite their ecological importance, salt ponds have received little scientific attention and remain poorly understood [10,11]. A thorough description of salt ponds is urgently needed to provide baseline information for wetland conservation efforts in the Caribbean and to establish frameworks for conservation and management protocols.

**Figure 1:**
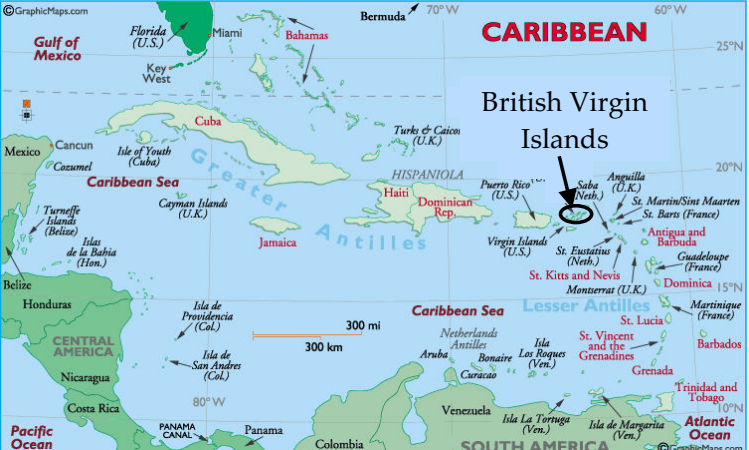
Map of the Caribbean region, showing the location of the British Virgin Islands. Image from http://worldatlas.com/webimage/countrys/carib.htm.

**Figure 2:**
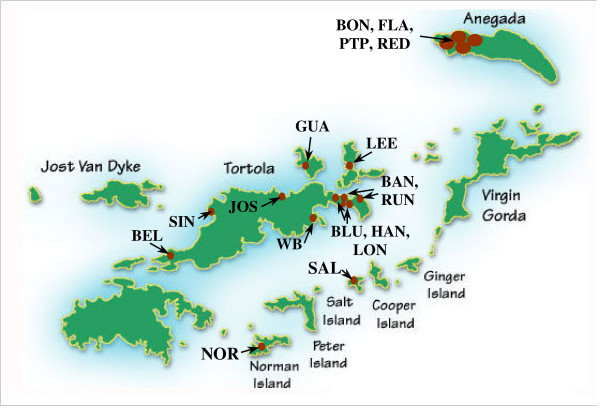
Map of the British Virgin Islands showing study site locations.

This study describes the physical characteristics of salt ponds in the British Virgin Islands (BVI), a small archipelago at the eastern end of the Greater Antillean island chain in the Caribbean (Figure 1). Nearly sixty salt ponds occur along the semi-arid coastlines of the BVI, which provided an opportunity to compare multiple habitats within a small geographical area. In this paper, we examine spatial and temporal variation in hydrology and salinity in 17 salt ponds (Figure 2; Table 1). Monitoring change, both cyclical and progressive, is essential for management and restoration of these vulnerable habitats [12,13]. This paper establishes a scientific baseline for such work.

**Table 1:** Key to abbreviated pond names, grouped by location

Abbreviation	Pond name	Location
**BON**	Bones Bight Pond	Anegada
**FLA**	Flamingo Pond	Anegada
**PTP**	Point Peter Pond	Anegada
**RED**	Red Pond	Anegada
**BAN**	Banana Wharf Pond	Beef Island
**BLU**	Bluff Bay Pond	Beef Island
**HAN**	Hans Creek Pond	Beef Island
**LON**	Long Bay Pond	Beef Island
**RUN**	Runway Pond	Beef Island
**LEE**	Lee Bay Pond	Great Camanoe
**GUA**	Guana Island Pond	Guana Island
**NOR**	Norman Island Pond	Norman Island
**SAL**	Salt Island Pond	Salt Island
**BEL**	Belmont Pond	Tortola
**JOS**	Josiah's Bay Pond	Tortola
**SIN**	Sinky Pond	Tortola
**WB**	Witches' Brew Pond	Tortola

### Salinity

Salinity is the predominant abiotic factor limiting aquatic communities in hypersaline water, and it influences both dissolved oxygen concentrations and temperature [14-16]. Salinity is known to fluctuate widely in shallow hypersaline water bodies because their high surface to volume ratio makes them especially sensitive to seasonal and shorter-term environmental changes [17]. Weather patterns control the concentration of salts by evaporation and dilution [18]. In coastal ponds, seawater input and flushing can also influence salinity [19-21].

We anticipated salinity fluctuations in BVI salt ponds similar to those reported for saline lakes and coastal salt ponds elsewhere in the world, and we expected the amplitude of these salinity fluctuations to be influenced both by weather patterns and by a pond's degree of hydrological connection with the sea.

### Hydrology

Salt ponds are dynamic ecological systems that evolve through a series of natural changes. Succession via sedimentation and changing hydrological conditions should occur in the salt ponds of the BVI, and, consequently, present-day ponds should represent stages in a natural hydrological progression, from near-marine systems to near-terrestrial systems. Similar processes have recently been described in Belize [22].

Factors that influence the hydrology of wetlands include precipitation, catchment size, groundwater flow, surface flow, permeability of sediments, and vegetation. Precipitation in the BVI is seasonal, with a rainy season from August through December. Salt ponds occur at the bottom of steep watersheds on all islands except Anegada, which is flat. They are not associated with estuaries, which are generally absent from dry Caribbean islands. Rainfall tends to run down hillsides over the surface rather than through the ground because the soil layer in the Virgin Islands is thin and the underlying rock has low permeability [23]. Surface flow occurs only after heavy rainfall, as there are no permanent rivers, creeks or streams in the BVI. Groundwater resources, which are limited to narrow alluvial valleys, scattered sand deposits and fractured volcanic rock, recharge at a rate of only 3 to 8 cm/yr [23]. Seawater, on the other hand, may enter salt ponds over the surface or through the ground because the berms that separate salt ponds from the sea are composed of permeable sediments, mainly sand and coral rubble. Seawater input is predicted to be an important force in controlling pond hydrology and salinity.

Four mangrove species, *Rhizophora mangle, Avicennia germinans, Laguncularia racemosa*, and *Conocarpus erectus*, make up the predominant vegetation along the shores of salt ponds. Their distribution among ponds in the BVI is described in [2] and will not be addressed here.

## Results

### Salinity

**Figure 3:**
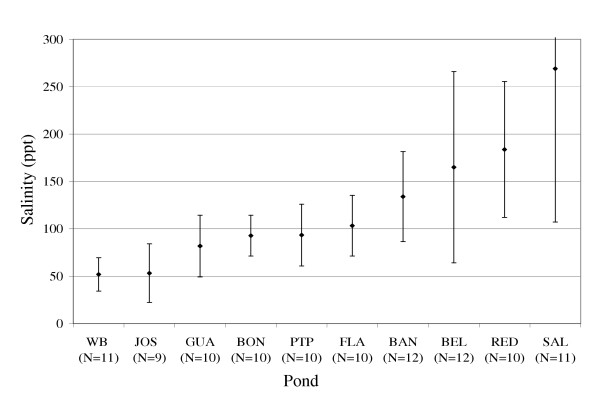
Mean of monthly salinity measurements during 1995. Error bars indicate one standard deviation above and below the mean.

Salinity fluctuated widely within ponds and mean salinities varied among ponds (Figure 3; Table 2). All of the 10 ponds sampled monthly over a full year were hypersaline, on average, with half having mean salinities below 100 ppt and half above (Figure 3). Nine of the 17 sampled ponds varied into mesosalinity (20 – 50 ppt [1]), and one, JOS, became hyposaline (< 20 ppt [1]) after sustained and heavy rainstorms. Lowest salinities were found in JOS and WB, and variability was lowest in WB.

**Table 2:** Salinities recorded outside of the period of regular monitoring (1995).

Salinity			
Pond	Mean & std dev.	Range	N
BON	71 ± 10	57 – 83	5
FLA	98 ± 26	70 – 130	6
MAN	79 ± 9	72 – 85	2
PTP	66 ± 16	48 – 80	3
RED	117 ± 36	66 – 160	5
BAN	90 ± 36	46 – 146	11
BLU	72 ± 32	41 – 110	3
LON	65 ± 30	40 – 130	8
RUN	84 ± 27	45 – 110	4
BEL	160 ± 83	65 – 305	12
JOS	46 ± 32	9 – 120	14
SIN	44 ± 8	35 – 51	3
WB	62 ± 21	47 – 77	2
GUA	72 ± 20	28 – 100	21
SAL	360	---	1

**Figure 4:**
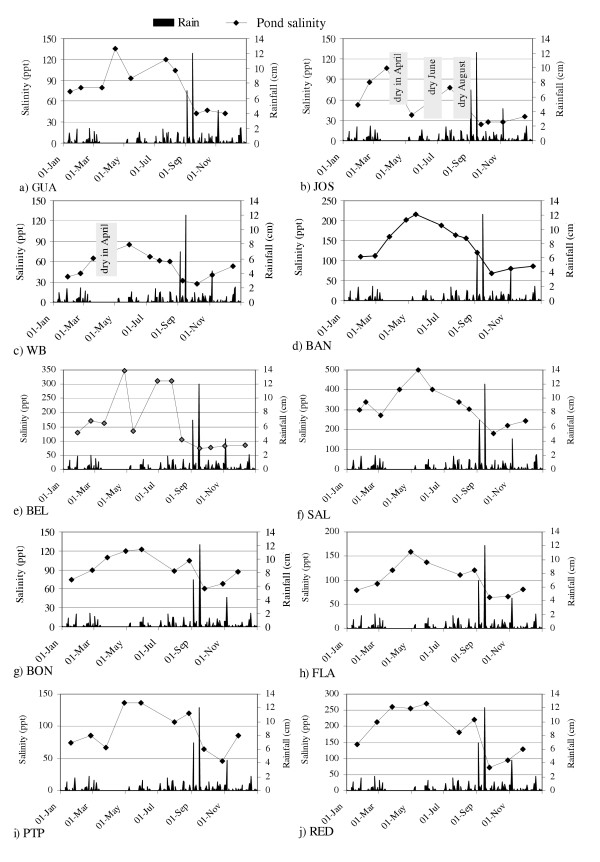
Salinity fluctuations in 10 ponds during 1995.

Highest salinities occurred in BEL and SAL. These ponds contained seawater seeps that continually supplied salts without introducing sufficient seawater to cause flushing. Sodium chloride crystallized over the bottom of SAL during extended dry periods in which pond salinities exceeded 300 ppt, the precipitation point of NaCl. At the same time gypsum crusts formed in BEL, which during dry periods experienced salinities between 175 ppt (the precipitation point for gypsum, CaSO_4_·2H_2_O) and 300 ppt (Figure 4). RED was also a highly saline pond, and most of its bottom was covered with a precipitated gypsum pavement. A seep may have existed at RED, but the perimeter of this pond was not fully explored during this study.

During 1995, salinity in all ponds increased between February and August, the driest part of the year, and it declined between August and November, the wettest part of the year (Figure 4). Dilution by rainfall was common to all ponds.

**Figure 5:**
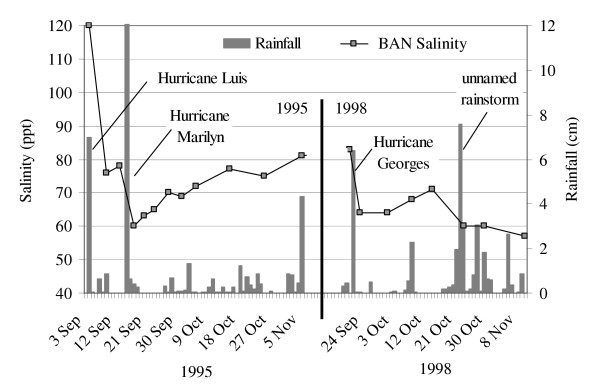
Salinity responses to hurricanes and other heavy rains in BAN.

**Figure 6:**
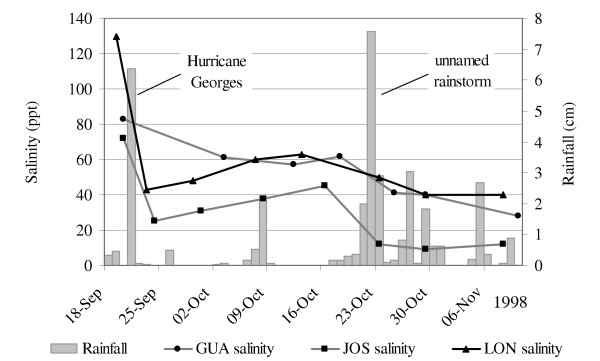
Salinity responses to hurricanes and other heavy rains in GUA, JOS and LON.

Dilution effects were more precisely illustrated by the hurricane monitoring data (Figure 5 &6). Hurricane Luis (Sept. 4^th^, 1995), with 7 cm of rain, caused the salinity in BAN to fall 37% in two days (Figure 5). Ten days later, 12 cm of rain fell during Hurricane Marilyn (Sept. 15^th^, 1995), bringing about a further 23% decline in salinity. Thus over ten days, the salinity of BAN was reduced by 50%, from 120 ppt to 60 ppt. Afterwards, the salinity increased slowly to 87 ppt by December.

Hurricane Georges (September 21^st^, 1998) affected BAN similarly (Figure 5). It brought 6.5 cm of rain, which diluted the salinity of BAN by 23%, from 83 to 64 ppt. Salinity gradually increased to 71 ppt by October 14^th^, but another 7.5 cm of rain fell between October 21^st ^and 23^rd ^and caused a further 15% dilution to 60 ppt. Continued rainfall through the following month maintained relatively low salinity in this pond.

Similar effects were seen in 3 other ponds monitored during Hurricane Georges (Figure 6). Salinity in GUA dropped by 27%, from 83 to 61 ppt; salinity in JOS dropped by 65%, from 72 ppt to 25 ppt; and salinity in LON dropped by 67%, from 130 ppt to 43 ppt. After Hurricane Georges, salinity in these ponds gradually increased until the October 21^st ^rains, which diluted GUA by 34%, from 62 to 41 ppt, diluted JOS by 73%, from 45 to 12 ppt, and diluted LON by 21%, from 63 to 50 ppt.

Pond water did not show salinity stratification except for short periods after rainfall. In GUA, 6.5 cm of rain (on 28 and 29 July, 2000) dropped the salinity of the surface water from 75 ppt to 47 ppt. Over the following 36 hours, surface and bottom salinity gradually approached equilibrium at 57 ppt. This pattern was repeated on a smaller scale after a rainfall of 0.18 cm on 19 August, 2000, when the surface water of GUA was diluted to 6 ppt less than bottom water (73 ppt), and equilibrium (71 ppt) was achieved after 9 hours.

### Ground water salinity

Salinity in groundwater wells at the vegetation/shore border of FLA averaged 81 ± 4 ppt on 9 August, 1998, while the pond salinity was 89 ppt. No halocline was detected in the groundwater wells. Ten meters behind the shore vegetation where the mangrove fringe began, groundwater salinity (46 ± 0.7 ppt) was closer to that of seawater.

**Figure 7:**
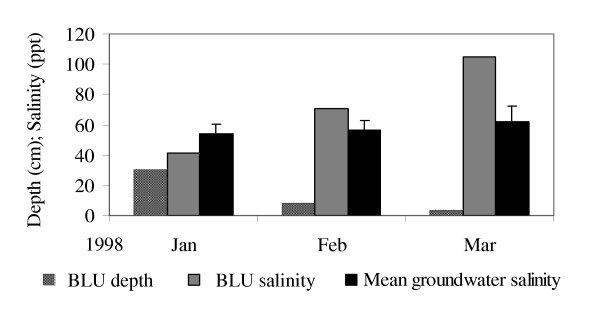
Groundwater salinity, pond salinity and pond depth at BLU, January – March, 1998.

At BLU, groundwater salinity averaged 54 ± 6 ppt on January 23, 1999. Groundwater salinity showed only a small increase to 62 ± 10 over the following 2 months, during which time the pond lost 27 cm in depth and gained 64 ppt in salinity via evaporation (Figure 7).

### Evaporation

The rate of long-term evaporation in BAN was 32 m^3 ^H_2_O day^-1^ha^-1^, between 26 March and 26 April, 1995. A corresponding salinity concentration of 125%, from 160 ppt to 200 ppt, was measured in BAN during this drought period.

Short-term evaporation rate in BAN in April, 2000 (Table 3), included a clear day and a cloudy day, was similar (32 to 45 m^3 ^H_2_O day^-1^ha^-1^) to the long-term evaporation rate measured in April, 1995. No salinity change was detected (± 1 ppt minimum detection limit). No evaporation occurred at night.

**Table 3:** Rates of evaporation in salt ponds.

Pond	Date (2000)	Time Interval	Weather	Depth Change (cm)	Evaporation (m^3^/d-ha)
BAN	17–18 Apr	16:30- 10:00	clear	-0.33	45
	20-Apr	9:30- 18:30	prt cloudy	-0.12	32
	13–14 Aug	21:30- 7:20	night	0	0
JOS	18-Apr	9:00- 16:50	clear	-0.5	150
	26-Jul	13:30- 17:40	prt cloudy	-0.24	140
	26-Jul	17:40- 21:40	prt cloudy	-0.09	54
	26–27 Jul	21:40- 7:00	clear	0	0

Short-term evaporation in JOS was very high (150 – 160 m^3 ^H_2_O day^-1^ha^-1^) during daylight hours in April and July (Table 3), corresponding with a 4 ppt increase in salinity (from 124 to 128 ppt) in one afternoon. Evaporation during evening hours was lower (53 m^3 ^H_2_O day^-1^ha^-1^), and no evaporation occurred during the night. By projecting these measurements over a whole day (proportioning daytime, dawn/dusk, and night hours), the whole-day mean evaporation rate was estimated at 64 m^3 ^H_2_O day^-1^ha^-1^.

### Inundation and seasonal depth fluctuations

**Figure 8:**
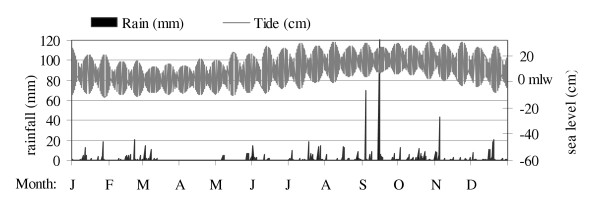
Rainfall and sea level fluctuations during 1995.

Salt ponds were found to differ in their inundated periods and in their degree of connection with the sea (Table 4). All ponds were shallow, with a ten-pond depth average of 29 ± 20 cm during 1995, though substantial variation existed among ponds (Table 4). Nearly half of the ponds dried completely for between 3 and 9 months annually (temporary ponds), while other ponds never dried (permanent ponds). All ponds received freshwater input via rainfall and runoff, but only eight of 17 ponds received direct seawater input. In the latter group, three types of seawater influence were found. Permanent sea connection, through a narrow channel, occurred in four ponds (BON, FLA, PTP & RED). Two other ponds (BEL & SAL) received seawater input via through-ground seeps that were visible at the shoreline. The remaining two ponds (LON & WB) were periodically connected with the sea during seasonal high tides (June through December, Figure 8).

**Table 4:** Hydrological characteristics of BVI salt ponds.

Pond	Inundation mo./yr.	Connection w/sea	**Tidal influence**^a^	Depth (cm)	Area (ha.)	Watershed (ha.)
**BON**	12	direct	3.5	46 ± 8	57	85
**FLA**	12	direct	0.93	26 ± 6	220	330
**PTP**	12	direct	0.75	12 ± 7	72	110
**RED**	12	direct	1.9	25 ± 7	110	170
**BAN**	12	none	0	39 ± 19	3.0	11
**GUA**	12	none	0.14	35 ± 16	1.9	18
**SIN**	12	none^b^	2.6	---	1.5	10
**BEL**	12	seep	0.70	23 ± 23	13	41
**SAL**	12	seep	---	24 ± 23	4.0	13
**HAN**	3	none	< 0.2	< 5	0.40	20
**LEE**	3	none	< 0.2	< 5	0.32	11
**NOR**	5	none	< 0.2	< 10	0.42	---
**JOS**	7	none	0	13 ± 16	9.0	150
**RUN**	7	none	0.82	---	4.7	13
**BLU**	8	none	1.7	---	3.6	40
**LON**	9	periodic	1.2	---	8.6	18
**WB**	9	periodic	0.19	21 ± 13	1.8	27

^a ^largest rise in water level (cm) recorded by tide-detection measurements. A pond was considered to be tidally influenced if the water level rise was > 0.2 cm.

^b ^possible underground connection

HAN, LEE and NOR held water only for short periods after rainfall, and they were inundated for a total of 3 to 4 months annually. Bottom sediments were sandy.

**Figure 9:**
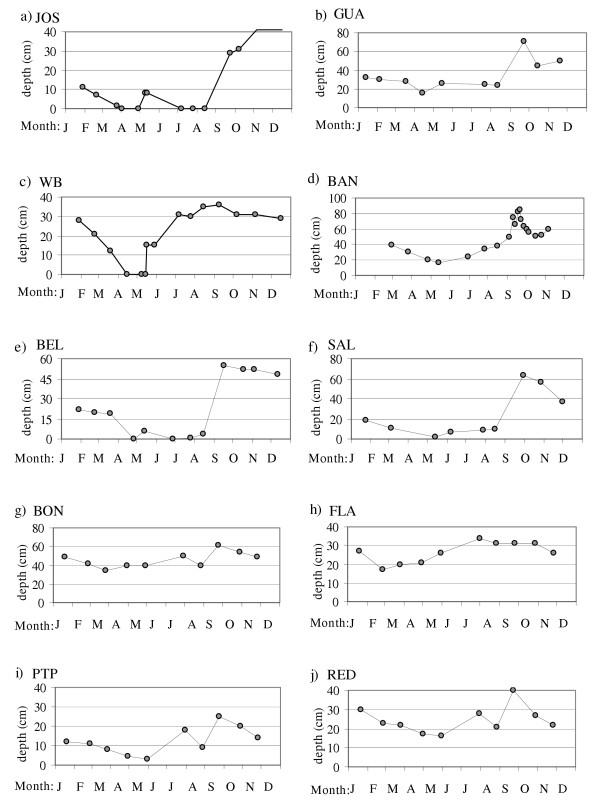
Depth fluctuations in ponds during 1995.

BLU, RUN and JOS (Figure 9a) were also greatly influenced by precipitation, but these ponds were continuously inundated for 7 months or more in most years. Bottom sediments in these ponds were predominantly sand, though in RUN and BLU patches of soft mud were also present.

GUA was a temporary (non-permanent) pond until 1990, after which saline water input via an overflow pipe and an effluent pipe from a nearby desalination plant resulted in permanent inundation. Regular input of seawater maintained a relatively stable water level in GUA for most of 1995, though water level dropped 12 cm during the April drought and rose 47 cm after hurricane rains in September (Figure 9b).

WB, with an initial depth of 28 cm in January, was completely dry by April (Figure 9c). The low-volume rains (1 cm) of May 7^th ^and 8^th^, which inundated JOS (Figure 9a), did not fill WB, presumably because of its smaller watershed area (Table 4). One week after these rains, a high tide of 23 cm above mean low water, which was the first of this magnitude for the year (Figure 8), filled WB to 15 cm (Figure 9c) when seawater breached a low point in the berm separating WB from the sea. Seawater connection through the resulting channel occurred regularly during high tides from June through December, when sea level was higher than earlier in the year (Figure 8). Water level in WB did not fluctuate greatly as long as the connection with the sea was maintained (Figure 9c). The hurricane rains of September had no effect on water level in WB. Direct connection with the sea during the hurricanes presumably allowed rapid equilibration of water level in WB by draining runoff to the sea.

Episodes of seawater inundation, similar to those in WB, were observed over several years (1998 to 2001) in LON, although depth was not monitored in this pond. Three concrete culverts, constructed to allow water to drain under the road that separated LON from the shore on the southern side of the pond, allowed seawater into LON in summer and fall when sea level was highest. The bottom of LON was composed mostly of sand with some patches of organic mud, while that of WB was composed of organic mud.

BAN and SIN were both permanently inundated but had no surface sea connection. The inundated portion of BAN shrunk to approximately 60% of its initial size and lost 22 cm in depth during the driest part of the year (Feb – May, Figure 8d). Hurricanes Luis and Marilyn raised the level of BAN by 26 cm and 13 cm, respectively. Water level fell 9 cm immediately after Hurricane Luis and 13 cm within days of Hurricane Marilyn, presumably via drainage through the sand and coral berm that separated BAN from the sea. The bottoms of both ponds were uniformly covered by organic mud, but unlike SIN, BAN supported benthic microbial communities that formed a firm gelatinous layer over the mud.

BEL and SAL were both permanently inundated and received small but constant seawater input through underground seeps, where water was observed trickling out through the shore sediments and into the ponds. Salinity of water emerging from these seeps was consistently near 40 ppt, which indicated a seawater origin. The berm in the seep area at SAL was composed of coral rubble, while the berm in the seep area at BEL was composed of organic and silty sediments. Depth fluctuations in these ponds were similar to one another and both ponds responded to rainfall and evaporation cycles (Figure 9e &9f). BEL and SAL became shallower and smaller during the drought period in March and April, 1995. The water level at BEL declined below the marked sampling area and thus exposed the depth reference point, hence the zero depth values for April, June, and July in Figure 9e. With increasing sea level between June and August, 1995, (Figure 8) greater seawater input through seeps at BEL and SAL was expected. Contrary to expectation, however, water level in these ponds did not rise in the summer. Rains from the September hurricanes, on the other hand, raised the water level more than 50 cm in both ponds. Bottoms these ponds were composed of thick organic mud deposits.

The Anegada ponds, BON, FLA, PTP and RED, were connected with one another and maintained a direct connection with the sea through a narrow channel near PTP. This channel measured 3.3 m wide and 37 cm deep at its narrowest point. Depth fluctuations in these ponds were generally similar to one another, and the ponds responded to seasonal changes in mean sea level more so than to rainfall and evaporation cycles (Figure 9g–j). Bottom sediments in this group of ponds varied from sand (PTP) to organic mud (BON). Sediments in FLA and RED were covered in most areas by a hard crust composed predominantly of gypsum crystals (CaSO_4_-2H_2_O).

### Tidal influence on pond water levels

BAN, GUA and JOS showed no evidence of tidal influence on pond water levels (Table 4). HAN, LEE and NOR were presumed to also be non-tidal because they remained dry, except for relatively short periods after rainfall, even during seasonally high tides from May through July.

Water levels in BLU, LON and RUN were influenced by tidal forces (Table 4), but this influence was not always detectable. Tide-induced fluctuations were detected on two out of four measurement periods in BLU, on two out of three measurements in RUN and in three out of four measurements in LON. The largest observed increase in BLU's water level occurred 10 hours after a high sea tide. In contrast, high water in LON and RUN generally occurred within one hour of the high tide. Tidal influence was not detected in LON or WB when, during periods of lower sea level, they lost direct connection with the sea. Tidal water level fluctuations were undoubtedly experienced in LON and WB at times of direct sea connection.

The water level in BEL rose substantially (0.7 cm) in response to a rising tide on 10 July, 2000, the only date on which it was sampled.

A water-level rise of 2.6 cm in SIN was higher than that measured in any other pond, and it occurred in response to a moderate high tide of only 14 cm above m.l.w.

All of the Anegada ponds (BON, FLA, PTP, RED) showed regular water level fluctuations in response to tidal cycles, and the timing these fluctuations closely followed that of tides.

## Discussion

BVI salt ponds were all shallow and mostly hypersaline, and their waters were generally well mixed. They exhibited large variations in salinity, both temporally and spatially, among ponds.

Rainfall controlled temporal salinity fluctuations (Figures 4, 5, 6), and it accounted for most, if not all, freshwater inputs to ponds. Groundwater appeared to be an unimportant source of freshwater input, as evidenced by the constant salinity of groundwater near ponds despite changing pond salinities (Figure 7). This finding agreed with observations from St. John, USVI [23]. We observed a decline in groundwater salinity with distance from the pond edge, a pattern typical of the interfaces between salt flats and mangroves and presumably caused by the greater influence of fresh water with distance from an evaporative basin [24].

Evaporation was an important force in all ponds, influencing both salinity and depth. Through-ground drainage of pond water to the sea did not normally occur, except when a pond was excessively full, as was observed in BAN after two consecutive hurricanes. Long-term and short-term methods of measuring evaporation yielded similar results, indicating that the mean evaporation rates in ponds during dry weather was between 32 and 64 m^3^H_2_O/day-ha, all (or nearly all) of which occurs during daylight hours. The maximum change in pond depth during dry weather was -0.5 cm/day, a value similar to the rate of evaporation (-0.6 cm/day) reported for a hypersaline lagoon in the Red Sea [25].

Pond water levels declined during dry periods, exposing large areas of pond sediments, while rainfall caused ponds to fill and expand. This effect was less pronounced in ponds with direct sea connections (Figure 9g–j). The buffering effects of seawater connection did not extend to salinity, as even the permanently sea-connected Anegada ponds became highly saline (>100 ppt) during dry periods (Figure 4g–j). This indicates that seawater entering the channel to the Anegada ponds did not flush concentrated waters out of the ponds. The phenomenon of hypersalinity despite communication with the sea has also been described in a Brazilian lagoon [20] and in a Saudi Arabian lagoon [25].

Salt ponds exhibited a range of hydrological differences. This variability allowed us to identify several groups of ponds, using inundation period and surface (visible) connection with the sea as primary descriptors. Below, the hydrological characteristics and characteristic salinity ranges are described for each of these groups.

1. Permanent ponds with direct sea connection (BON, FLA, PTP, RED):

These ponds, all situated on the flat coral island of Anegada, formed the largest wetland system in the BVI. They were inter-connected in addition to being connected with the sea through a narrow channel. Water levels in ponds with permanent direct sea connection were controlled by sea level fluctuations and by rainfall and evaporation. Salinity was variable but generally high (mean annual salinities ranged from 93 to 184 ppt; Figure 3). Rainfall and evaporation appeared to be far more important in forcing salinity fluctuations than was seawater flushing.

2. Permanent ponds with seawater seeps (BEL, SAL):

A constant seep of seawater trickled into these ponds through the shore sediments. Water levels were determined primarily by rainfall and evaporation, though small daily fluctuations in water level corresponded with tidal cycles. Ponds with seawater seeps experienced some of the highest salinities of all ponds (mean annual salinities ranged from 165 to 269 ppt; Figure 3). Gypsum and sodium chloride were deposited by precipitation in these ponds during the dry season.

3. Temporary ponds with periodic direct sea connection (LON, WB):

Ponds with periodic direct sea connection were filled by sea overwash during seasonally high tides between May and December (Figure 8). During these periods of sea connection, water level was controlled solely by tidal fluctuations. When tides were low, sea communication was broken, and then water levels were controlled by rainfall and evaporation cycles. Salinity fluctuations in these ponds were similar in magnitude to those in other temporary ponds. However, seawater flushing during the dry season reduced pond salinities, causing the salinity fluctuations in these ponds to differ in timing from those in other temporary ponds (Figure 4).

4. Permanent ponds with no surface sea connection (BAN, SIN):

Two ponds with rather different hydrologies are included here. Water level in BAN was influenced by rainfall and evaporation but not by tidal fluctuations. SIN, in contrast, exhibited large tidal fluctuations (Table 4). Unlike BAN, SIN maintained a relatively constant area of inundation throughout the dry season. Salinity in BAN was generally high and variable (135 ± 48 ppt; Figure 3), while that of SIN was consistently near seawater (35 – 51ppt; Table 2). In all of these respects, BAN was more similar to other ponds than SIN was.

SIN's unique hydrology, characterized by high-amplitude tidal fluctuations, permanent inundation and near-seawater salinity, suggests that this pond maintained underground connection with the sea, probably through its coral berm. Such hydrology is similar to that of the anchialine ponds of Bermuda, which maintain sea connection through underground caves [21,26].

Consequently, this category probably represents two distinct types of permanent ponds: those with underground sea connection (e.g. SIN) and those with no sea connection (e.g. BAN).

5. Temporary ponds without surface sea connection (BLU, HAN, JOS, LEE, NOR and RUN):

Inundation period in this group of ponds ranged from three to eight months per year. Inundation was controlled by rainfall and evaporation. Seasonally high tides did not force water into these ponds. Recorded salinities ranged from hyposaline (for brief periods after hurricane rains) to three times the seawater salinity (Table 2). In half of these ponds (BLU, JOS, RUN), tidal influence on water level, once inundated, was detectable, suggesting that their bottoms were at or below sea level. The other half of these ponds (HAN, LEE and NOR) were the smallest and shallowest ponds studied, and they had the shortest inundation periods (three to five months).

These hydrologically distinct groups of ponds reflect a trend from high connectivity with the sea to complete isolation from the sea. This trend is also apparent when the geomorphologies of bays and lagoons are considered. Completely open bays, or bights, lie at one extreme, followed by bays that are partially restricted by shallow coral reefs. Where mangroves colonize the exposed tops of these coral reefs, sediment accretion by mangrove roots leads to the construction of berms, which restrict wave energy within lagoons. Salt ponds lie at the other extreme, where a berm completely (in most cases) separates the inland water body from the sea.

**Figure 10:**
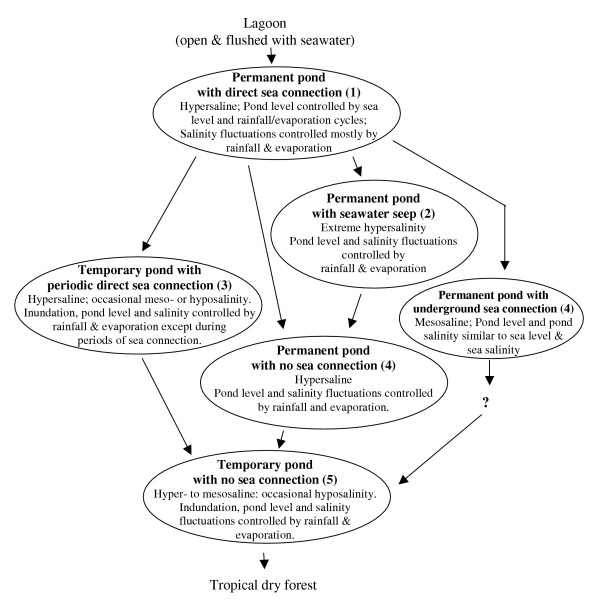
Proposed path of natural transformation of a lagoon through stages of salt pond and finally to dry forest. Numbers in brackets refer to the hydrological categories described in the Discussion section.

The existing hydrologic variability observed among BVI salt ponds suggests a pattern of geologic evolution from shallow coastal marine waters to salt ponds and eventually to dry land. BVI salt ponds thus provide present-day examples of natural wetland transformations that have previously been described by Dix *et al*. [27] in their geologic analysis of marine sediments from a Bahamian salt pond. A possible route through the observed stages of salt ponds is diagrammed in Figure 10. A pond with direct sea connection, depending on its geomorphic and sediment characteristics, might evolve into any one of four different types of ponds: 1) a permanent pond with underground sea connection, 2) a permanent pond with a seawater seep, 3) a permanent pond with no sea connection, or 4) a temporary pond with periodic direct sea connection. Of these, a permanent pond with a seawater seep might transform into one with no sea connection as the seep becomes blocked by sediments; a permanent pond with no sea connection may, in turn, become a temporary pond as it collects sediments until its bottom lies above sea level; and a temporary pond with periodic direct sea connection may become one with no sea connection as the berm through which sea connection is established is stabilized by mangrove roots and accreted sediments. The fate of a permanent pond with underground sea connection to become a temporary pond with no sea connection is unclear, as this type of pond was unique in our data set. Regardless, senescing ponds should all reach the stage of a temporary pond with no sea connection, and as these ponds fill with erosional sediments and as salts are washed out by rainfall, dry forest trees will encroach along the borders into the former pond.

## Conclusion

BVI salt ponds were hypersaline and showed dramatic salinity fluctuations in response to seasonal rainfall and evaporation patterns. Hydrological variation ranged from permanently inundated ponds with direct sea connection to those fully isolated from the sea and retaining water only after rainfall. Observed variability allowed a classification based on shared hydrological characteristics. This classification appeared to reflect a geologic evolution from open lagoons through several types of salt ponds and finally to salt flats, into which tropical dry forest may intrude (Figure 10).

Identification of representative stages of salt pond evolution will assist in policy and decision making, particularly because external factors such as watershed runoff, sedimentation, infilling and eutrophication, as well as sudden or severe weather events and urban coastal development, will influence salt pond hydrology and salinity. Understanding whether changes are reversible, whether resources are available to reverse or restrain trends, and what will be the implications of non-reversal or slowing of successional changes are all management decisions that need to be made for these habitats. The patterns of variability and succession described here can be used to guide such management decisions.

Anthropogenic activities greatly accelerate the evolutionary processes in salt ponds, thereby rapidly degrading the ecological benefits of salt ponds. Advantages for flood alleviation, sediment retention, shore-line stability, migratory bird populations and others are reduced as salt ponds become sites for landfill, dumping or building (all observed recently in the BVI). The consequent pressures on neighbouring ecosystems, such as silting of bays and reefs, justify further detailed analysis, monitoring and management of salt ponds.

## Methods

A territory-wide survey of BVI salt ponds was conducted in 1995, and a subset of ten ponds, distributed across 5 islands, was selected for monitoring. Salinity and depth were recorded monthly from January through December of 1995. Logistical difficulties prevented two sampling visits to Anegada (June & December; four ponds) and one visit to Guana (December; one pond). Additional salinity measurements were recorded intermittently between 1991 and 2001. Mean pond salinities compared in this paper were calculated from the 1995 data exclusively, because they reflect a full annual cycle of salinity fluctuations in each pond.

Salinity changes during and after hurricanes were monitored intensively (two to three samples weekly) in one pond in 1995 and in four ponds in 1998. Subsequent sampling, performed between 1998 and 2001, focussed on describing hydrological variation among ponds.

Physical characteristics, including pond area, surface connection with the sea, composition of bottom sediments, period of inundation and watershed area were described in 17 ponds (Table 1; Figure 2). Tidal water movements were measured in all but four ponds, three of which were dry for the majority of each year.

Each pond was mapped and measured (area, perimeter and nearest distance to the sea) using the British Virgin Islands National GIS database (2001–2002) provided by the BVI Conservation and Fisheries Department. These dimensions included only the regularly inundated, non-vegetated portion of each pond.

Watersheds were measured by tracing over a GIS contour map the area of hillside that drained into each pond. Pond areas were included in watershed dimensions. Contours for Anegada and Norman Island were not available. The watershed areas for four ponds on Anegada, which is flat (maximum elevation 9 m), were approximated as 1 1/2 times the area of each pond, an estimate based on the overall geography of the island. The watershed area of NOR was not estimated. Watershed area was used as a relative measure of the potential effect of rainwater entering each pond.

Observations of geomorphology, surface connection with the sea, berm structure, sediments, and surrounding land use were noted while walking the perimeter of each pond.

Pond inundation (presence or absence of standing water) was noted during site visits and at any other time when such observations were possible (e.g. travelling within view of a pond). Inundation periods are reported as the maximum number of months per year that each pond was continuously inundated. Water depth was measured using a weighted measuring tape at permanent sampling stations at each of 10 ponds during 1995 only.

Tidal influence on water level in ponds was determined by tracking the position of the water's edge and converting this lateral movement to vertical movement by triangulation from the shore slope (the shoreline tracking method, detailed in [2]). Because pond shores were nearly flat, this method was highly sensitive to water movements. A water level drop of only 1 cm, for example, would expose half a meter of shoreline. Wind and resulting small waves introduced a standard error of ± 0.026 cm depth, based on measurements from 10 different days in a non-tidal pond (JOS). The maximum change caused by a moderate wind (approximately 20 knots) blowing towards the sampling area corresponded with a 0.14 cm rise in water level. To avoid classifying a pond as tidally influenced in error, we used a 0.2 cm increase in water level as the minimum threshold for positive determination of tide-driven water movement in ponds (tidal influence). Water level measurements were conducted on clear days with wind speeds of 20 knots or less. Measurements immediately following rain showers were excluded from this analysis. Declining water levels were not used to ascertain the presence of tidal influence because ebbing water was not distinguishable from evaporative water loss.

For ponds that did not initially show tidal fluctuations, water level measurements were repeated at least three times, including during at least one tidal cycle in which the sea rose higher than 25 cm above mean low water (m.l.w). The mean level of high tide during a typical year (1998) was 20 cm above m.l.w [28].

The timing and amplitude of sea tides were determined by Walker's (1992) DOS program for worldwide tide predictions [28], using St. Thomas, U.S. Virgin Islands, as the closest reference point. No time correction was used, as the reference point was within 50 km of all ponds except the Anegada ponds, which were within 90 km of St. Thomas. Sea level was monitored during February, 1998, using a graduated stake in shallow water at Bluff Bay, Beef Island. The observed timing of high and low tides was approximately equivalent to those predicted by Walker's program. Mean sea levels shown in this paper were calculated as consecutive 5-day means of the difference between m.l.w. and the daily high and low tides.

Water salinity was measured *in situ *using a hand-held refractometer with automatic temperature compensation (Vista, model A366ATC). Samples having salinities beyond the range of the instrument (100 ppt) were proportionally diluted with distilled water before the final measurement. Instrument resolution was 1 ppt and error was ± 2 ppt; however, when dilution was necessary, error increased to a maximum of 5% of water salinity.

Salinity stratification was assessed in BAN on 13 August (16:30 and 20:45) and 14 August (7:00 and 11:50), during dry weather. It was also assessed in GUA after rainfall between 29 July and 1 August, 2000, (6 samples) and between 19 August and 20 August (5 samples).

Groundwater salinity was monitored monthly from January through March of 1998 in 4 constructed groundwater wells (1.0 to 1.3 m deep) approximately 30 cm above the vegetation line around the perimeter of BLU. Another 4 wells (0.7 to 0.9 m deep, 0.5 to 10 m landward of vegetation line) were sampled at FLA on 9 August, 1998.

Precipitation data were supplied by local residents at two locations. Other long-term rainfall data were not available in the BVI.

A drought period between 14 March and 6 May, 1995, during which no rainfall was recorded anywhere in the BVI, provided an opportunity for evaluating long-term evaporation rates in salt ponds. The drought-period change in pond depth and salinity in BAN was used to calculate long term evaporation. BAN was chosen for this analysis because it was completely isolated from seawater influence (including tidal effects) and it retained water throughout the dry season. The resulting evaporation model assumed that the quantity of water lost or gained through the ground was insignificant when compared with evaporative water loss. In fact, pond levels monitored by the shoreline tracking method (described above for detection of tidal influence) were constant at night, indicating that through-ground drainage was minimal at best. Evaporation was expressed as the volume of water lost per day per hectare of pond, as these units allowed for the comparison of volume losses between ponds of different sizes.

A second method for measuring evaporation assessed small changes in water level over a number of hours using the shoreline tracking method. Only data from ponds in which no tidal influence was detected, BAN and JOS, were used for these calculations of short-term evaporation.

## Competing interests

The author(s) declare that they have no competing interests.

## Authors' contributions

L. Jarecki conducted the field work, analysis and reporting.

M. Walkey served as advisor to the project in all stages.
